# Hydrogen Gas Attenuates Toxic Metabolites and Oxidative Stress-Mediated Signaling to Inhibit Neurodegeneration and Enhance Memory in Alzheimer’s Disease Models

**DOI:** 10.3390/ijms26146922

**Published:** 2025-07-18

**Authors:** Sofian Abdul-Nasir, Cat Tuong Chau, Tien Thuy Nguyen, Johny Bajgai, Md. Habibur Rahman, Kwon Hwang-Un, In-Soo You, Cheol-Su Kim, Bo Am Seo, Kyu-Jae Lee

**Affiliations:** 1 Department of Global Medical Science, Wonju College of Medicine, Yonsei University, Wonju 26426, Republic of Korea; abdulnasirsofian62@gmail.com (S.A.-N.); chaucattuong2014@gmail.com (C.T.C.); thuyttienw@gmail.com (T.T.N.); 2 Department of Convergence Medicine, Wonju College of Medicine, Yonsei University, Wonju 26426, Republic of Korea; johnybajgai@yonsei.ac.kr (J.B.); globaldreamer1990@yonsei.ac.kr (M.H.R.); cs-kim@yonsei.ac.kr (C.-S.K.); 3 Organelle Medicine Research Center, Wonju College of Medicine, Yonsei University, Wonju 26426, Republic of Korea; 4 Department of Laboratory Medicine, Wonju College of Medicine, Yonsei University Wonju 26426, Republic of Korea; 5 GOOTZ Co., Ltd., Yuljeongro, 247 beon-gil, Yangju-si 11457, Republic of Korea; kwon@mygootz.com (K.H.-U.); igootz@naver.com (I.-S.Y.); 6 Department of Biochemistry, Wonju College of Medicine, Yonsei University, Wonju 26426, Republic of Korea; 7 Natural Product Research Center, Korea Institute of Science and Technology, Gangneung 25451, Republic of Korea

**Keywords:** Alzheimer’s disease, oxidative stress, neuroinflammation, neurodegeneration, apoptosis, molecular hydrogen, astrocytes and urea cycle

## Abstract

Alzheimer’s disease (AD) is a neurodegenerative condition in which amyloid-beta (Aβ) plaques trigger oxidative stress (OS) and neuroinflammation, causing memory loss. OS and neurodegeneration can also be caused by reactive astrocytes, thereby promoting AD via toxic metabolite accumulation in the astrocytic urea cycle. However, the effect of molecular hydrogen (H_2_) on this cycle remains unknown. Therefore, we investigated whether H_2_ treatment could reduce OS-induced neurodegeneration and memory loss. 5xFAD (*n* = 14) and wild-type (*n* = 15) mice were randomized into four groups and treated with either 3% hydrogen gas (H_2_) or vehicle for 60 days. Cognitive behaviors were evaluated using the Morris water maze and Y-maze tests. In addition, we used biochemical assays to measure ammonia and hydrogen peroxide (H_2_O_2_) levels in the hippocampi of the mice and AβO-treated primary mouse astrocytes. Aβ, γ-aminobutyric acid (GABA), and the expression of inflammatory markers were evaluated using immunohistochemistry (IHC) and quantitative real-time polymerase chain reaction (qRT-PCR). We observed that H_2_ treatment significantly prevented cognitive deficits, oxidative stress, the accumulation of toxic metabolites, and the increase in inflammatory markers in 5xFAD mice. These results suggest that H_2_ therapy can mitigate toxic metabolites in the astrocytic urea cycle, thereby reducing neurodegeneration and memory loss in AD.

## 1. Introduction

Memory impairment and neurodegeneration are pathophysiological hallmarks of Alzheimer’s disease (AD), which predominantly manifests in people aged 65 years and older and constitutes a severe public health burden. In AD, amyloid-beta (Aβ) plaques, i.e., the significant deposition of insoluble Aβ fibrils and tau tangles, cause oxidative stress (OS), inflammation, and apoptosis when they accumulate in the brain; thus, they are key markers of AD pathogenesis [1,2,3]. OS is implicated in the onset of neurodegenerative disorders, including AD, Parkinson’s disease, and mild cognitive impairment. It can destroy neuronal lipid membranes, cause DNA damage-related cell death, and stimulate further Aβ accumulation via protein oxidation mechanisms [4]. These effects cause neuronal damage, culminating in the memory loss associated with AD [5] and mitochondrial dysfunction [6,7,8], and resulting in defective cytochrome oxidase activity and altered Ca^2+^ signaling [9,10,11]. Similarly, Bax, a pro-apoptotic marker, can activate caspases in the mitochondria (e.g., caspase-3, -8, and -9), contributing to neurodegeneration through apoptosis [7]. OS can trigger neuroinflammation via the nuclear factor kappa B (NF-κB) and mitogen-activated protein kinase (MAPK) nexus, causing neurodegeneration and AD [12].

Neurodegeneration and memory loss can also result from reactive astrocytes [13]. Astrocytes become “double-edged swords” by providing neuroprotection and becoming neurotoxic as OS and nitrosative stresses increase [14]. This neurotoxicity is due to the increased accumulation of toxic metabolites, which enhances the reactivity of astrocytes. Toxic metabolites such as ammonia activate the astrocytic urea cycle and trigger the synthesis of putrescine, hydrogen peroxide (H_2_O_2_), and gamma (γ)-aminobutyric acid (GABA) [15,16]. In the context of AD, the intracellular accumulation of amyloid-β protein (Aβ) can be cleared by the autophagy–lysosomal pathway, releasing amino acids such as aspartate, which enters the astrocytic urea cycle and is converted to arginosuccinate by arginosuccinate synthetase 1. The arginosuccinate is then transformed into arginine via the catalytic activity of arginosuccinate lyase. Arginase 1 (ARG1) converts arginine to ornithine, and urea is released as a waste product. Ornithine undergoes decarboxylation through ornithine decarboxylase 1 (ODC1) activity to produce putrescine, which goes through a series of enzymatic transformations to produce H_2_O_2_ and ammonia as side products and GABA as the final product. The ammonia produced undergoes N-acetylation with glutamate, forming carbamoyl phosphate. Ornithine transcarbamylase (OTC) then converts carbamoyl phosphate to citrulline. Citrulline can also be converted to arginosuccinate, thereby repeating the cycle [13,17,18]. H_2_O_2_, a key reactive oxygen species (ROS), and GABA contribute to neurodegeneration and memory impairment, respectively [18]. The key enzymes responsible for producing these toxic metabolites are ARG1, ODC1, and OTC [19].

Ju et al. established the molecular mechanisms underlying the bifunctional role of reactive astrocytes, which comprise a beneficial urea cycle-active pathway that detoxifies metabolites of Aβ breakdown and a harmful cycle that increases ODC1 expression, which facilitates toxic H_2_O_2_, ammonia, and GABA accumulation [18]. Elevated astrocyte reactivity can lead to the overexpression of glial fibrillary acidic protein (GFAP) levels. The overexpression of GFAP, an emerging diagnostic marker of AD, signals astrocyte death, also known as astrogliosis [16].

Despite extensive studies on the pathophysiology of AD, there is currently no cure for AD [20]. Therapeutic options, such as cholinesterase inhibitors [21], memantine, lecanemab, and donanemab, can only delay the progression of AD [22,23]. Recently, molecular hydrogen (H_2_) has been effective against many diseases, including neurodegenerative disorders [17,24,25,26]. H_2_ effectively reduces OS, inflammation, and apoptosis [27,28,29]. H_2_ has a small molecular size, making it easy for H_2_ to penetrate cell membranes and cross the blood–brain barrier, exerting effects on the central nervous system [17,24,25,26]. In neurodegenerative disorders, including AD, H_2_ therapy has been shown to attenuate OS via selective reduction in ROS and modulating inflammatory and apoptotic signaling pathways [27,30]. Specifically, hydrogen-rich water has been linked to suppressing the NLRP3 inflammasome, an essential component of the innate immune response, which has been implicated in AD [31]. In addition, H_2_ therapy has been shown to modulate brain-derived neurotrophic factor and estrogen receptor β, which are critical for synaptic plasticity and survival of neurons [32]. Further, in a chemically induced mild cognitive impairment model, H_2_ has been reported to increase spatial memory and reduce OS, Aβ, Bax, and cleaved caspase-7 levels [29]. Despite H_2_’s therapeutic benefits, its effect on the astrocytic urea cycle metabolic pathway remains to be explored.

Thus, this study aimed to investigate whether H_2_ treatment decreases the accumulation of toxic metabolites in the astrocytic urea cycle, thereby slowing neurodegeneration and memory loss. We hypothesized that H_2_ could downregulate the accumulation of toxic metabolites in the urea cycle, reducing neurodegeneration and memory impairment in the 5 familial AD (5xFAD) mouse model, as assessed using behavioral tests, biochemical assays, Western blot, immunohistochemistry (IHC), immunocytochemistry (ICC), and gene expression analysis.

## 2. Results

### 2.1. H_2_ Inhalation Attenuates Memory Impairment in 5xFAD Mice

**Figure 1:**
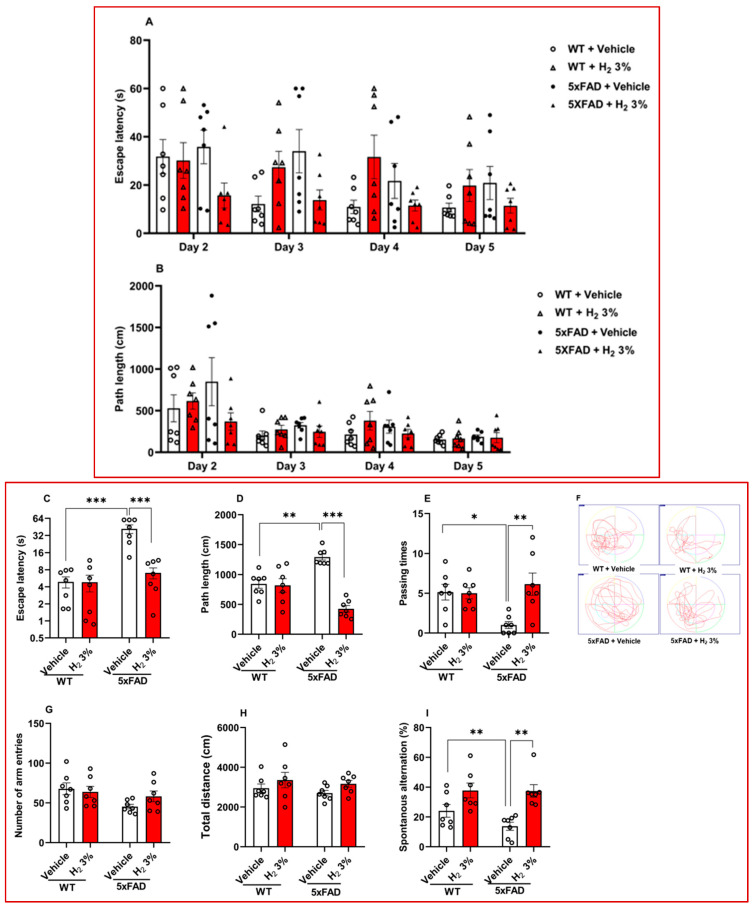
Mice behavioral test results. Morris water maze results, including (**A**) escape latency during trial (days 2–5), (**B**) path length during trial (days 2–5), (**C**) escape latency for test trial (day 6), (**D**) path length for test trial (day 6), (**E**) passing time for test trial (day 6), and (**F**) swimming pattern. Y-maze test results: (**G**) number of arm entries, (**H**) total distance, and (**I**) spontaneous alternation. WT and 5xFAD mice were treated with 3% H_2_ (*n* = 7 in each group). Significant differences were set at * *p* < 0.05, ** *p* < 0.01, and *** *p* < 0.001. H_2_, molecular hydrogen; 5xFAD, 5 familial Alzheimer’s disease; WT, wild type.

We assessed the effect of H_2_ treatment on hippocampal-dependent spatial learning and memory impairment in 5xFAD mice using the Morris water maze and Y-maze tests. We analyzed escape latency, path length, and passing time from the Morris water maze test. During tests conducted on days 2–5 (hidden platform), we observed that the H_2_-treated 5xFAD mice showed a 1.4-fold decrease in escape latency time and a 2.0-fold decrease in path length compared with the vehicle-treated mice (Figure 1A,B). In the test trial on day 6, the escape latency time of the H_2_-treated 5xFAD mice decreased by a factor of 6.0 (*p* < 0.001; Figure 1C) and path length decreased by a factor of 3.0 (*p* < 0.001; Figure 1D), compared with the vehicle-treated mice; furthermore, the H_2_-treated 5xFAD mice traveled into the target quadrant (i.e., passing time) 6.0-fold more often than the vehicle-treated mice (*p* < 0.01; Figure 1E), as further illustrated by swimming patterns shown in Figure 1F. For the Y-maze test, the arm entry frequency (Figure 1G), total distance (Figure 1H), and the percentage of spontaneous alternation (Figure 1I) were analyzed. The percentage of spontaneous alternation markedly increased by 2.7-fold (*p* < 0.01) in the H_2_-treated 5xFAD mice compared with the vehicle-treated mice (Figure 1I).

### 2.2. H_2_ Reduced OS and Decreased Neuroinflammation in AβO-Treated Astrocytes

**Figure 2:**
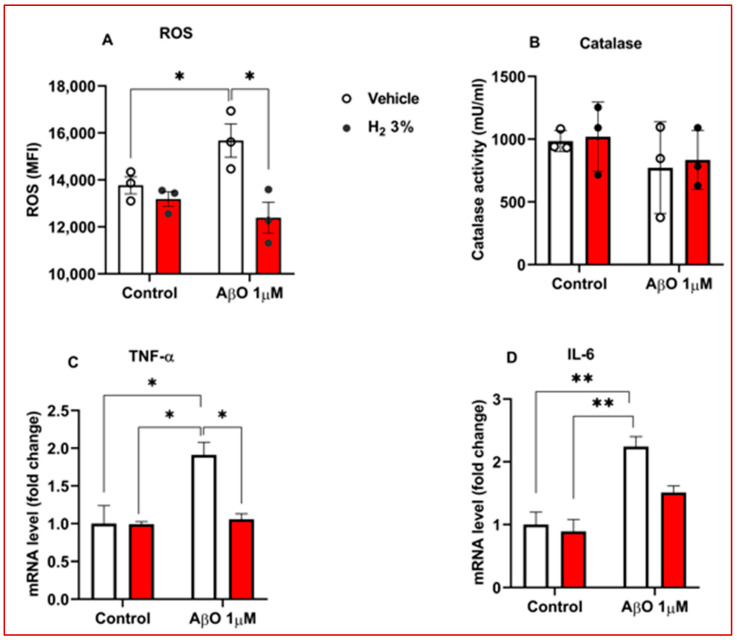
Effects of H_2_ treatment on OS and inflammatory markers in in vitro AD model. (**A**–**D**) Bar charts of ROS assay, catalase assay, mRNA level of TNF-α, and IL-6 in Aβ-induced astrocytes (2 × 10^5^ cells/well) treated with H_2_ and vehicle (*n* = 3 for each group), respectively. H_2_, molecular hydrogen; IL-6, interleukin 6; OS, oxidative stress; ROS, reactive oxygen species; TNF-α, tumor necrosis factor-alpha. Solid circles and red bars represent H_2_ treatment and hollow circles in white bars represent vehicle treatment. Statistical significance was set at * *p* < 0.05, ** *p* < 0.01.

Increased oxidative stress and neuroinflammation are characteristic pathophysiological features of Alzheimer’s disease [12]. To evaluate the therapeutic effect of H_2_ in modulating these pathological markers, we assessed intracellular ROS levels, catalase antioxidant enzyme activity, and pro-inflammatory cytokines in AβO-induced astrocytes. H_2_ treatment significantly attenuated ROS production in AβO-stimulated primary mouse astrocytes, showing a 1.2-fold reduction compared with vehicle (*p* < 0.05; Figure 2A). In addition, catalase activity increased by 20% following H_2_ treatment, although this increase was insignificant (Figure 2B). To further investigate the anti-inflammatory potential of H_2_ in the AβO-stimulated primary mouse astrocytes, we quantified the expression of TNF-α and IL-6, the common neuro-inflammatory markers, using qRT-PCR. We observed a significant reduction in TNF-α mRNA levels (*p* < 0.05; Figure 2C) compared with vehicle, but the decrease in IL-6 expression was not significant (Figure 2D).

### 2.3. H_2_ Reduced OS and Neuroinflammation in 5xFAD Mice

**Figure 3:**
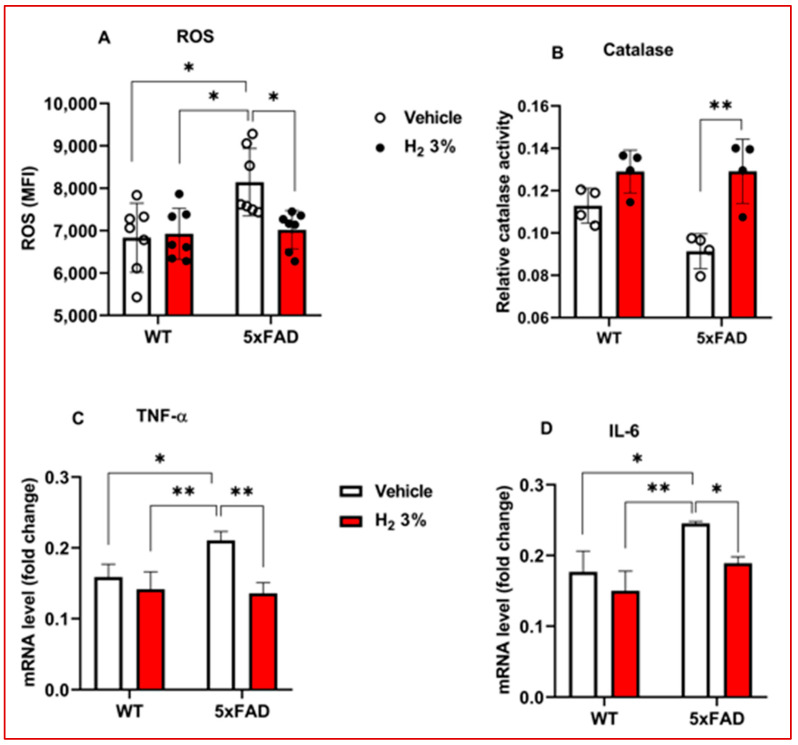
Effects of H_2_ treatment on OS and inflammatory markers in vivo AD model. (**A**–**D**) Bar charts of ROS assay (*n* = 7), catalase assay (*n* = 4), mRNA levels of TNF-α, and IL-6 in mice treated with H_2_. WT (*n* = 3) and 5xFAD (*n* = 3). 5xFAD, 5 familial Alzheimer’s disease; H_2_, molecular hydrogen; TNF-α, tumor necrosis factor-alpha; IL-6, interleukin 6; OS, oxidative stress; ROS, reactive oxygen species. Solid circles and red bars represent H_2_ treatment and hollow circles in white bars represent vehicle treatment. Statistical significance was set at * *p* < 0.05, ** *p* < 0.01.

The effects of H_2_ treatment on intracellular ROS levels, catalase antioxidant enzyme activities, and pro-inflammatory cytokines were also assessed in the hippocampal tissue from 5xFAD mice. We detected a marked effect, with a 1.2-fold decrease in ROS levels (*p* < 0.05; Figure 3A) and a 1.4-fold increase in catalase activity (*p* < 0.01; Figure 3B) compared with the vehicle-treated group. H_2_ treatment markedly suppressed TNF-α and IL-6 mRNA expression by 1.6-fold (*p* < 0.01) and 1.3-fold (*p* < 0.05), respectively, as compared to the vehicle-treated group (Figure 3C,D).

### 2.4. H_2_ Improved Amyloid Pathology and Reduced Toxic Metabolite Accumulation in 5xFAD Mice and AβO-Induced Primary Astrocytes

**Figure 4:**
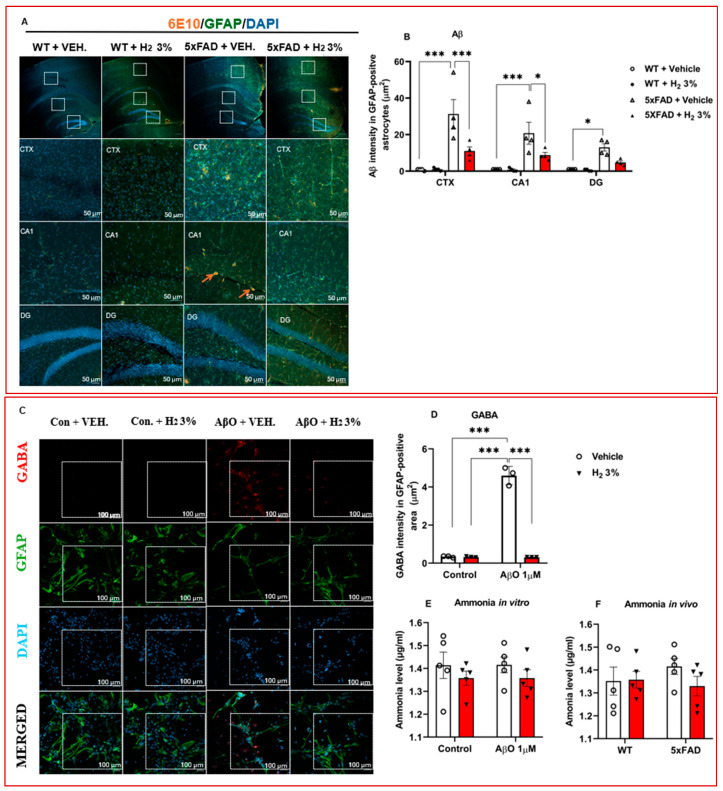
Toxic metabolite levels after H_2_ treatment. (**A**) IHC confocal image of mice brain sections. (**B**) Plot of amyloid-beta signal intensity (*n* = 4). (**C**) ICC image for GABA signal detection in astrocytes. (**D**) Graph of GABA intensity (*n* = 4). (**E**,**F**) Graphs show ammonia concentration for in vitro and in vivo assays. Statistical significance was set at * *p* < 0.05, *** *p* < 0.001. H_2_, molecular hydrogen; CTX, cortex; CA1, cornu ammonis 1; DG, dentate gyrus; 5xFAD, 5 familial Alzheimer’s disease; GABA, γ-aminobutyric acid; ICC, immunocytochemistry; IHC, immunohistochemistry; VEH, vehicle; 6E10 positive indicates amyloid plaques. Arrows show amyloid plaques, and white boxes represent section detected under the microscope.

Amyloid plaques can activate the astrocytic urea cycle, causing the accumulation of toxic metabolites, resulting in astrocyte reactivation, neurodegeneration, and memory loss [18]. To evaluate the effects of H_2_ treatment on amyloid pathology, GFAP expression, and toxic metabolites (H_2_O_2_, ammonia, and GABA) accumulation, we performed IHC, ICC, and biochemical assays using hippocampal sections and lysates of mice and astrocytes. We first analyzed the ROS levels reflecting H_2_O_2_ and found a significant decrease in the H_2_-treated AβO-induced astrocytes and 5xFAD, compared to their vehicle-treated controls (Figure 2A and Figure 3A). Next, we performed IHC and observed that Aβ plaque accumulation in GFAP decreased by 2.8-fold in the cortex (CTX) (*p* < 0.001), 2.4-fold in the cornu ammonis 1 (CA1) (*p* < 0.05), and 2.7-fold in the dentate gyrus (DG), as shown in Figure 4A,B. Similarly, the GABA levels were markedly lower (*p* < 0.001) in the AβO-induced astrocytes treated with H_2_ than in those treated with vehicle; this was confirmed with subsequent ICC analyses (Figure 4C,D). Ammonia concentrations were measured and observed to have decreased minimally in vitro, as shown in Figure 4E,F.

## 3. Discussion

In this study, using 5xFAD transgenic mice and AβO-induced primary astrocytes, we examined the effects of H_2_ treatment on astrocytic urea cycle metabolites and enzymes after 60 days of 3% H_2_ exposure in the mice and 3 h in the astrocytes. The main findings of the analysis were as follows: (1) We observed that 5xFAD mice treated with H_2_ exhibited significant memory improvement, as shown by the significantly higher percentage of passing time in the Morris water maze test and higher spontaneous alternation percentage in the Y-maze test (Figure 1E,I, respectively). These indicate that 3% H_2_ inhalation significantly improved spatial memory and learning in 5xFAD mice compared with the vehicle. (2) OS (i.e., ROS and catalase activity) and inflammatory markers decreased with H_2_ treatment in both AβO-induced astrocyte cultures and 5xFAD mice, with significant differences between the treatment and vehicle groups (Figure 2A–D and Figure 3A–D). These findings imply that H_2_ therapy lowers OS and inflammation by decreasing the H_2_O_2_ levels via its antioxidant activity and decreasing TNF-α and IL-6 gene expression via an anti-inflammatory mechanism. Thus, suggesting that H_2_ induced neuroprotective effects by mitigating OS and downregulating inflammatory cytokine expression in AβO-induced astrocytes and 5xFAD mice. The observed reductions in ROS and pro-inflammatory mediators highlight the therapeutic potential of H_2_ as an agent targeting redox imbalance and neuroinflammation in AD progression. (3) The levels of toxic metabolites were significantly lower in the treatment group than in the vehicle group, with Aβ plaques (represented by the number of 6E10-positive signals) and GABA showing significant differences (Figure 4A–E). These data demonstrate that H_2_ treatment attenuated toxic metabolite accumulation, including Aβ, H_2_O_2_, and GABA, which were markedly reduced after H_2_ treatment compared with the vehicle. These suggest that astrocyte function was enhanced after H_2_ treatment due to reduced astrogliosis, denoted by the decrease in the GFAP level.

The antioxidant defense system is key to H_2_’s efficacy, which is modulated upstream by nuclear factor erythroid 2-related factor 2 (Nrf2) [33]. Nrf2 translocates to the nucleus under OS conditions, upon Keap1 modification, and binds to the promoter regions of antioxidant response elements. The binding drives the transcription and subsequent expression of many antioxidant enzymes, including catalase, glutathione peroxidase, and superoxide dismutase [34]. Through these mechanisms, Nrf2 prevents cell damage from reactive oxygen and nitrogen species and maintains redox homeostasis [35]. Studies on the medical applications of H_2_ have increased in recent years, with different methods of H_2_ administration in pre-clinical models and clinical subjects. These include inhalation, ingestion (dissolved in water), intravenous injection, and gel application to the skin [36,37]. The concentration of H_2_ in various studies varies. Approximately 1–4% of H_2_ is deemed safe [36], and 3% of inhaled H_2_ has been reported to improve cognitive and diffusion tensor imaging scores in patients with AD [38]. Additionally, 2% inhalation has been observed to reduce the risk of AD by downregulating the biomarker levels in healthy adults. These studies have advanced our understanding of the therapeutic efficacy of H_2_ [36,39]. However, the variations in dosage and routes of administration make these findings difficult to compare [36,38,39]. Moreover, these studies lacked randomization [38], and to date, no one has investigated the mechanisms by which H_2_ modulates the toxic metabolites associated with the astrocytic urea cycle in AD models.

Recent studies [18,40] have proposed ODC1 as a suitable therapeutic target for AD; however, it has been shown that difluoromethylornithine, an ODC1 inhibitor, requires high dosage and has toxic side effects [41,42]. Furthermore, another study reported no significant differences in ODC1 inhibition between difluoromethylornithine treatment and control [43]. A recent study proposed that tonic GABA may target reactive astrocytes in brain diseases; however, further evidence is needed to support this hypothesis [44]. Our study showed improved learning and memory after treating 5xFAD mice with 3% H_2_, which may have resulted from reduced OS and inflammatory markers, consistent with the results of earlier studies [37,38,39,45].

H_2_O_2_, a primary ROS, is produced as a toxic secondary metabolite in the astrocytic urea cycle; H_2_ is a scavenger of H_2_O_2_, thereby reducing neurodegeneration. The reduction in OS via H_2_O_2_ scavenging likely contributed to the observed decrease in the Aβ levels (Figure 4B), consistent with the findings of previous studies [12,46,47]. Additionally, accumulating Aβ in the brain can trigger OS and neuro-inflammatory responses in astrocytes, resulting in neuronal dysfunction and apoptosis [12]. The high levels of OS induce protein oxidation, resulting in Aβ formation [5]. Our study showed a decrease in Aβ, OS (illustrated by the decrease in ROS with the increase in catalase activity), and inflammatory markers such as TNF-α and IL-6, suggesting a decline in neurodegeneration; this enhances intraneuronal signal transmission and prevents the worsening of AD, as shown by memory improvement in mice (Figure 1E,I). The reduced OS also implies a decrease in co-localization of Aβ in the GFAP area (Figure 4A,B), suggesting that astrocyte death was reduced, improving its function. Furthermore, GABA, an inhibitory neurotransmitter that is produced in the final step of the astrocytic urea cycle, causes memory impairment in AD [17]. Our results showed a decrease in the GABA levels (Figure 4D), contributing to the memory improvement observed in the H_2_-treated 5xFAD mice.

However, the absence of metabolomic and proteomic profiling of specific urea cycle intermediates and enzymes and upstream markers of the antioxidant defense pathway, such as Nrf2, restricts our capacity to broadly evaluate the complete metabolic impact of H_2_ treatment. Future investigations incorporating integrated proteomic and metabolomic analyses are essential to describe the full scope of the H_2_-mediated modulation of the astrocytic urea cycle. Nevertheless, our findings indicate that H_2_ suppresses the accumulation of toxic urea cycle metabolites, thereby mitigating neurodegeneration and memory loss in an AD model.

## 4. Materials and Methods

### 4.1. Animal and Cell Culture Models

All animals were managed according to the Institutional Animal Care and Use Committee guidelines of Wonju College of Medicine, Yonsei University, which granted ethical approval for this study (ethical approval no: YWC-240423-2)**.** Male and female mice (*n* = 29) at 5 weeks old were used, comprising transgenic 5xFAD mice (*n* = 14) with amyloid precursor protein (APP) and presenilin 1 (PSEN1) overexpression [48,49] and C57BL/6 wild-type (WT) mice (*n* = 15). The 5xFAD transgenic mice (RRID: MMRRC_034848-JAX) were obtained from The Jackson Laboratory (Bar Harbor, ME, USA; Stock No: 008730). Hemizygous strains were bred by mating transgenic mice with F1 C57BL/6 mice, and their genotypes were confirmed using polymerase chain reaction (PCR). The information on primers used for the genotyping is shown in Table A2. The mice were kept under a 12:12 h light/dark cycle in cages, with food and water access. The treatment groups (WT, *n* = 8; 5xFAD, *n* = 7) were administered H_2_ (3%) by inhalation using an H_2_-producing device (GOOTZ Co., Ltd., Yangju-si, Republic of Korea) for 60 days. In contrast, the vehicle treatment groups (WT, *n* = 7; 5xFAD, *n* = 7) received normal ventilation. One death was recorded in the WT H_2_-treated group. After 60 days, behavioral tests were performed, the mice were sacrificed, and their brains were removed and preserved for further analysis. Brain lysates were prepared by homogenizing 20 mg of tissue, and protein concentration was determined using a BCA assay. Each sample was normalized to 5 mg/mL of protein.

For in vitro experiments, we prepared primary cortical astrocyte cultures using 1-day-old pups of C57BL/6 mice. Cerebral cortices were dissected, minced, and separated into single cells. We then plated cells in 0.1 mg/mL poly-D-lysine-coated plates. The cells were cultured in Dulbecco’s modified Eagle’s medium (Gibco, Paisley, UK) containing 4.5 g/L glucose, L-glutamine, sodium pyruvate, 10% heat-inactivated fetal bovine serum (SIGMA-ALDRICH, Carlsbad, CA, USA), and 5% penicillin–streptomycin. The cultured cells were maintained under humid conditions at 37 °C, with 5% CO_2_. The cells were washed with fresh media, floating cell debris was removed, and the medium was replaced after three days [50].

The primary astrocyte culture was then grown in separate 6-well plates (2 × 10^5^ cells/well) for further experiments. AβO enhances AD progression [3]; thus, to evaluate the effects of H_2_ treatment in AβO-induced astrocytes, the cells in one of the plates were induced with 1 µM AβO (rPeptide, Watkinsville, GA, USA) for 5 days and placed in a FLUXLUK cell treatment chamber connected to the H_2_-producing device (GOOTZ Co., Ltd., Yangju-si, Republic of Korea) for a total of 3 h at 30 min intervals, with H_2_ concentration set at 3%. In the other plate, the cells were treated with NH_4_OH, i.e., vehicle, for a similar duration. AβO was prepared by adding 1% ice-cold ammonium hydroxide (NH_4_OH) to Aβ monomers aliquoted into 1.5 µL microtubes and stored at −80 °C until needed.

The treatment duration differed between the in vivo and in vitro experiments due to the differences in biological context and experimental constraints. In the mouse model, a prolonged 60-day exposure was necessary to mimic the chronic progression of AD-like pathology. In contrast, primary astrocytes in vitro are more vulnerable to environmental stress, particularly under non-standard incubation conditions such as those in the FLUXLUK chamber. Thus, to avoid inducing cellular stress or death, H_2_ exposure was limited to short intervals totaling 3 h, which is a commonly used approach in similar in vitro antioxidant treatment studies. This short-term exposure was sufficient to assess the protective or regulatory responses of astrocytes to AβO and H_2_ treatment.

### 4.2. Behavior Test

Memory impairment is a hallmark of AD [20]. To assess the effect of H_2_ cognitive behavior in 5xFAD mice, we used the Morris water maze and Y-maze to evaluate spatial learning and short-term memory [51,52]. All behavior tests were double-blind; unblinding was only performed after the data were analyzed. The Morris water maze test was conducted as published by Bromley-Brits et al. [53]. Briefly, each mouse was carefully positioned in the first quadrant to swim and find the visible platform. Mice that could not locate the platform after 60 s were gently led to it and left for 10 s. This procedure was repeated for each mouse across all quadrants for five days. The platform was hidden from days 2 to 5 to assess spatial learning. On day 6, the platform was removed entirely, and individually, the mice were positioned in the quadrant farthest from the target quadrant to explore for 60 s. The escape latency and path length were determined from the average of five trials for each mouse. A camera (Panlab Harvard Apparatus, Holliston, MA, USA, Panlab SMART video tracking Smart 3.0, Harvard Apparatus) tracked and recorded the movements of the mice, and subsequent data analysis was performed to evaluate their performance. On the sixth day, escape latency, path length, and passing time were analyzed to assess spatial memory and learning abilities.

In the Y-maze test, the mice were placed individually at the maze’s center (Jeongdo B&P Co. Ltd., Seoul, Republic of Korea) with three identical arms (120° from each other) to explore for 8 min. All four limbs of the mouse were within the arm before entry was recorded. We used video tracking as described above and analyzed the frequency of arm entries and spontaneous alternations [52].

### 4.3. Assessment of Toxic Metabolite Levels

Ju et al. [18] reported that astrocytes remove toxic metabolites such as ammonia, GABA, and H_2_O_2_ via the urea cycle under normal conditions. However, these metabolites accumulate in AD, triggering OS, neuroinflammation, and apoptosis, resulting in neurodegeneration and memory loss. We investigated the effects of H_2_ on the levels of these toxic metabolites through biochemical assays, IHC, and ICC.

#### 4.3.1. ROS and Antioxidant Enzyme Assay

The intracellular ROS levels, specifically H_2_O_2_, were measured using 2,7-dichlorofluorescin diacetate (DCF-DA) (EMD Millipore Corp., Beijing, China). Briefly, in vivo, 10 µL of hippocampal lysates (5 mg/mL) and 100 µL of 20 µM DCF-DA were aliquoted into a 96-well plate. The plate was then incubated for 30 min in the dark. After the incubation, a DTX-880 microplate reader (Beckam Counter Inc., Fuller, CA, USA) was used to measure fluorescence at 488 nm excitation/525 nm emission. For in vitro assays, primary astrocytes were cultured and induced with 1 µM AβO (rPeptide, Watkinsville, GA, USA) in 6-well plates (2 × 10^5^ cells/well) for 5 days, then treated with H_2_ (3%) or 1% NH_4_OH for 3 h and washed two times in 1× phosphate-buffered saline (PBS) (Lonza, Walkersville, MD, USA); fluorescence detection was conducted as described above.

Antioxidant enzyme activity was quantified using the Amplex Red Catalase Assay Kit (Invitrogen, Carlsbad, CA, USA). Catalase breaks down H_2_O_2_, producing water and oxygen, thereby preventing cell damage [54]. Thus, 25 µL of each sample (5 mg/mL) and control were added separately in respective wells on the plate. Next, 25 µL of 40 µM H_2_O_2_ solution was added, and the content was kept at approximately 25–37 °C for 30 min. We then added 50 µL of 100 µM Amplex Red reagent/0.4 U/mL horseradish peroxidase, and the plate was incubated for 30 min at 37 °C. Subsequently, the absorbance was measured at 560 nm using a SpectraMax ABS Plus microplate reader (Molecular Devices, San Jose, CA, USA). The change in fluorescence was calculated as the fluorescence of the no-catalase control minus the fluorescence of the samples.

#### 4.3.2. Ammonia Assay

To quantify ammonia concentration in AβO-induced primary astrocyte cultures and mouse brain tissues, we used the Ammonia Assay Kit (Sigma-Aldrich, St Louis, MO, USA) per the product protocol. The cells (2 × 10^5^ cells/well) or hippocampal lysate (5 mg/mL) was made into a homogenous mixture using distilled water and centrifugation. The supernatant was optimized to a pH of 7–8, and 100 µL of this supernatant was placed in cuvettes. The L-glutamate dehydrogenase solution was added to the cuvettes, mixed thoroughly, and incubated for 5 min at 18–35 °C. Then, the absorbance was measured at 340 nm using a microplate reader, as described above.

#### 4.3.3. IHC and ICC

To determine whether 3% H_2_ treatment decreased Aβ accumulation in the 5xFAD mice hippocampi, we performed IHC as follows. The left hemispheres of excised mice brains were fixed with paraformaldehyde (4%) overnight at 4 °C and dehydrated in sucrose (30%) for 2 days. A cryostat was used to slice 30 µm coronal sections of the hippocampus. The sections were stored at 4 °C; they were washed in PBS and immersed in a blocking buffer, which comprised 5% goat serum in 0.1 M PBS with 0.3% Triton X-100 for 1 h. Next, the slices were incubated in the blocking buffer containing primary antibodies (Dako, Carpinteria, CA, USA) at 4 °C, amidst agitation, until the following day. They were then washed three times to remove unbound antibodies using PBS with 0.03% Triton X-100, and incubation with secondary antibody was carried out at room temperature for 1 h. Washing was repeated, and 4′,6-diamidino-2-phenylindole (DAPI) in PBS (1:1500 dilution) was added; the sections were subsequently mounted with a fluorescence mounting medium (Cell Signaling Technology, Danvers, MA, USA) and allowed to dry. Fluorescent images were captured using a confocal microscope and a Zeiss LSM880 microscope (Zeiss, Jena, Germany). Images were processed using the ImageJ software, version 1.41 (National Institutes of Health, Bethesda, MD, USA). The primary antibody and secondary antibody information are shown in Table A2.

Immunocytochemistry was used to analyze the effect of H_2_ on GABA accumulation. Briefly, AβO-induced astrocytes treated with 3% H_2_ or vehicle were fixed with paraformaldehyde (4%) for 15 min and washed repeatedly (three times) in cerebrospinal fluid. PBS containing 0.2% Triton X-100 was used to permeate the fixed cells and blocked for 1 h at room temperature. The cells were then incubated with a mouse anti-GABA primary antibody (Millipore, Bayswater, VIC, Australia) for 1 h at 4 °C. Next, the cells were incubated with a secondary antibody and DAPI (Vectashield^®^, Newark, CA, USA) for 40 min. After every incubation, three washes were performed with PBS containing 0.1% Triton X-100. Imaging was performed and analyzed as in IHC above.

### 4.4. RT-qPCR Analysis of Inflammatory Genes

To evaluate how H_2_ affects the inflammatory cytokine gene expression, we performed RT-qPCR on AβO-induced astrocytes (2 × 10^5^ cells/well) and mice hippocampal lysates (5 mg/mL). First, we extracted RNA from astrocytes and hippocampal lysates using the easy-BLUE^TM^ Total RNA Synthesis Kit (iNtRON Biotechnology, Seongnam-si, Republic of Korea). The extracted RNA (normalized to 1 µg) was converted to cDNA using the PrimeScript^TM^ 1st strand cDNA Synthesis Kit (Takara, Seoul, Republic of Korea). After cDNA library preparation, we conducted RT-qPCR as described by Kwak et al. [55]. Briefly, samples were prepared in triplicate, with a final volume of 10 µL comprising 1 µL of 10 pM primer, 2 µL of cDNA, 5 µL of SYBR Green PCR Master Mix (Applied Biosystems, Warrington, UK), and 2 µL of RNase-free water; these samples were analyzed in QuantStudio 6 Flex Real-Time PCR System (Applied Biosystems^®^, Marsiling Industrial Estate Road 3, Singapore). GAPDH mRNA was used as a reference, and fold change was determined using 2^−∆∆CT^. The primers used in this experiment are listed in Table A1.

### 4.5. Statistical Analysis

We used the GraphPad Prism software (version 8; GraphPad Software, La Jolla, CA, USA) to analyze data. Two-way analysis of variance with multiple comparison tests (Tukey’s post hoc test) was used to detect differences, with a significance level set at *p* < 0.05. Data from experimental samples are represented as mean and SEM. Individual mice or batches of cultured cells are represented as individual data points and samples, unless otherwise specified in the figure legends.

## 5. Conclusions

Our findings demonstrate that H_2_ treatment effectively attenuated the accumulation of toxic metabolites, OS, and inflammatory markers in both AβO-induced astrocytes and the 5XFAD transgenic mouse model of AD. Moreover, 3% H_2_ administration reduced the levels of H_2_O_2_, a toxic by-product of the astrocytic urea cycle, and GABA, thereby promoting neuroprotection. These biochemical improvements were associated with enhanced cognitive performance, as evidenced by improved learning and memory in 5xFAD mice. As we currently understand it, this is the first study to explore the therapeutic potential of H_2_ in targeting toxic metabolite accumulation within the astrocytic urea cycle in AD models. These findings suggest that H_2_ may represent a novel and promising strategy for mitigating neurodegeneration by modulating astrocyte-mediated metabolic dysfunction in AD. Nevertheless, detailed proteomic and metabolomic analyses are required to unravel the effects of H_2_ on the intermediates and enzymes of the astrocytic urea cycle.

## Data Availability

The original contributions presented in this study are included in the article. Further inquiries can be directed to the corresponding authors.

## List of Abbreviations

Aβ		Amyloid-beta
AβO		Amyloid-beta oligomer
AD		Alzheimer’s disease
ARG1		Arginase 1
CA1		Cornu Ammonis 1
CTX		Cortex
DCF-DA		2,7-dichlorofluorescein diacetate
DG		Dentate gyrus
5xFAD		5 Familial Alzheimer’s disease
GABA		Aminobutyric acid
GFAP		Glial fibrillary acidic protein
H_2_		Molecular hydrogen
H_2_O_2_		Hydrogen peroxide
ICC		Immunocytochemistry
IHC		Immunohistochemistry
IL-6		Interleukin 6
ODC1		Ornithine decarboxylase 1
OS		Oxidative stress
OTC		Ornithine transcarboxylase
ROS	Reactive oxygen species	
RT-qPCR		Quantitative real-time polymerase chain reaction
TNF-α		Tumor necrosis factor-alpha

## Appendix A

**Table A1:** List of primers.

Gene	Forward Primer Sequence	Reverse Primer Sequence
*APP/PS1*	5′-ACC CCC ATG TCA GAG TTC CT-3′	5′-CGG GCC TCT TCG CTA TTA C-3′
*TNF-α*	5′-TGTGCTCAGAGCTTTCAACAA -3′	5′-CTTGATGGTGGTGCATGAGA-3′
*IL-6*	5′-GCTACCAAACTGGATATAATCAGGA-3′	5′-CCAGGTAGCTATGGTACTCCAGAA-3′

**Table A2:** List of antibodies.

Antibody	Host	Application	Source	Dilution
Anti-Aβ	Pig	IF	MILLIPORE,	1:500
Anti-GABA	Mouse	IF	MILLIPORE	1:500
Goat anti-rabbit IgG	Goat	IF	Invitrogen	1:500
Goat anti-mouse IgG	Goat	IF	Invitrogen	1:500
Goat anti-guinea pig IgG	Goat	IF	Invitrogen	1:500
